# Cystic metastasis of prostate cancer: A case report

**DOI:** 10.1097/MD.0000000000013697

**Published:** 2018-12-14

**Authors:** Bei Zhang, Bingyang Bian, Lirong Bi, Zhuo Wang, Yang Zhao, Jiping Wang

**Affiliations:** a Department of Radiology; b Department of Pathology, First Hospital of Jilin University, Changchun, China.

**Keywords:** case reports, diagnosis, neoplasm metastasis, prostatic neoplasms

## Abstract

**Rationale:**

Prostate cancer often metastasizes (most commonly to the pelvic lymph nodes and axial skeleton); however, metastases to the pelvic cavity as a solitary mass are unusual. While metastatic prostate cancer is unconventional in pelvic cavity, cystic pelvic lesions are even more scarce. Accurate identification of cystic metastasis can be helpful in management of prostate cancer.

**Patient concerns:**

A 64-year-old male was admitted to our hospital due to urethral irritation symptom and dysuria.

**Diagnosis:**

In addition to prostate cancer, abdominal computed tomography (CT) scanning and magnetic resonance imaging (MRI) of the prostate revealed that a cystic mass was located at right pelvic cavity. Histopathological examination diagnosed the pelvic cystic mass as metastasis from prostatic cancer. Immunohistochemistry results demonstrated Calretinin (+), D2-40 (−), Ki-67 (10%+), Vimentin (−), CK-pan (+), CK5/6 (−), WT-1 (−), PSA (+), SALL4 (−), Villin (−), CK20 (−), CK7 (−), PAX-8 (−), and TTF-1 (−).

**Interventions:**

The cystic mass was removed. Primary cancer of the prostate was reserved as well. After discharge, the patient underwent in a two-year androgen deprivation therapy (ADT) treatment.

**Outcomes:**

After 13 months of discharge, no disease progression was found in the patient.

**Lessons:**

Although cystic prostate cancer is rare, the occurrence possibility should be considered when cystic lesions are accompanied with prostate cancer.

## 1 Introduction

According to a previous study, prostate cancer stands in the first place during the estimated new cases, as well as being the second cause of death of the most common cancers in the United States.^[1]^ In China, the largest increase in incidence was observed for cancers of the prostate, cervix, and thyroid among women between 2000 and 2012.^[2]^

The bone and lymph nodes have been recognized as the most common sites of metastases from prostate cancer; however, atypical metastases, including brain, liver, and lung metastases are rare.^[3]^ The death of patients with prostate cancer mainly occurs due to distant metastasis. Therefore, an early diagnosis of atypical metastasis can improve the prognosis of patients.^[3]^

Cystic metastasis of prostate cancer has scarcely been reported. Cystic metastasis of prostate cancer is an uncommon phenomenon that cannot be distinguished from other diseases, such as cystic lymph nodes. Diagnosis of cystic metastasis of prostate cancer is a main challenge, because it lacks imaging features.^[4]^ Lack of knowledge about the cystic metastasis of prostate cancer may lead to inappropriate medical measures as well.^[4]^

In the present report, a case with cystic metastasis of the prostate cancer proved by pathology was assessed. This article can be an awareness regarding raising of diversity of an atypical prostate cancer metastasis.

### 1.1 Presentation of the case

The case was a 64-year-old Chinese man with urethral irritation symptom, including voiding difficulty, in addition to the pain of the root of the penis for 3 months. He was admitted to The First Hospital of Jilin University (Changchun, Jilin province, China) on March 24, 2017. In the immediate aftermath of the irritation symptom, the increased interrupt urination sprang up. During urination, distraction pain in the root of the penis appeared. These symptoms have not been either treated in hospitals, or were previously examined. Despite the history of diabetes, no other diseases combined. The patient had a history of diabetes for 11 years. The level of blood glucose was satisfactory under the regular insulin therapy. The patient was active in a civil service company with typical Asian population eating habits and regular eating schedule. The patient had no history of smoking and alcohol abuse as well. While the family history of other diseases could not be verified, it was clear that the patient had no family history of prostate cancer.

In physical examination, there was no bulge in the suprapubic bladder area. No lump could be touched in the abdomen. There was also no tenderness that could be detected in the abdomen neither. Digital rectal examination (knees to chest position) was conducted as follows: prostate was moderately enlarged, shallow median groove, surface uneven, and a hard and nontender nodule could be palpated in the right lobe. Observation of the patient's urination included urinary urgency and frequency of night urination exceeded twice. During urination, there was voiding dysfunction, urine thinning, and interruption of urine flow.

**Figure 1:**
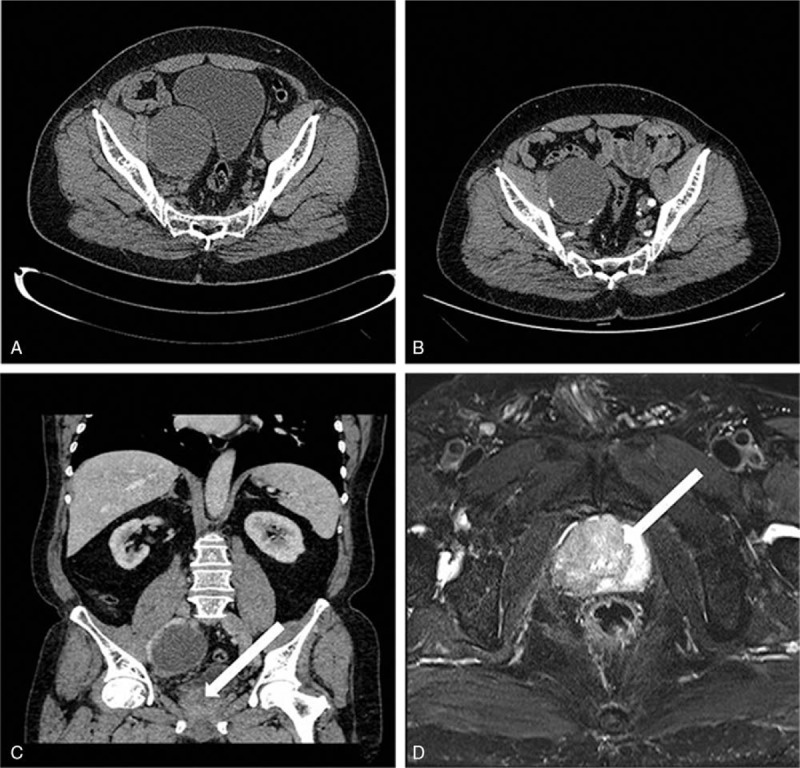
Abdominal CT scanning and MRI of the prostate. (A, B) The nonenhanced and arterial phase of abdominal CT axial scanning shows that a watery low density was located adjacent to the right iliac vessel. There was no obvious enhancement of the lesion in the arterial phase as well. (C) Venous phase of abdominal CT scanning exhibited that there was no significant enhancement in the cystic lesion of right lower abdomen. The enhancement of right part prostate is slightly obvious. (D) Fat-suppressed T2-weighted sequence presents low signal in the right portion of prostate. CT = computed tomography, MRI = magnetic resonance imaging.

**Figure 2:**
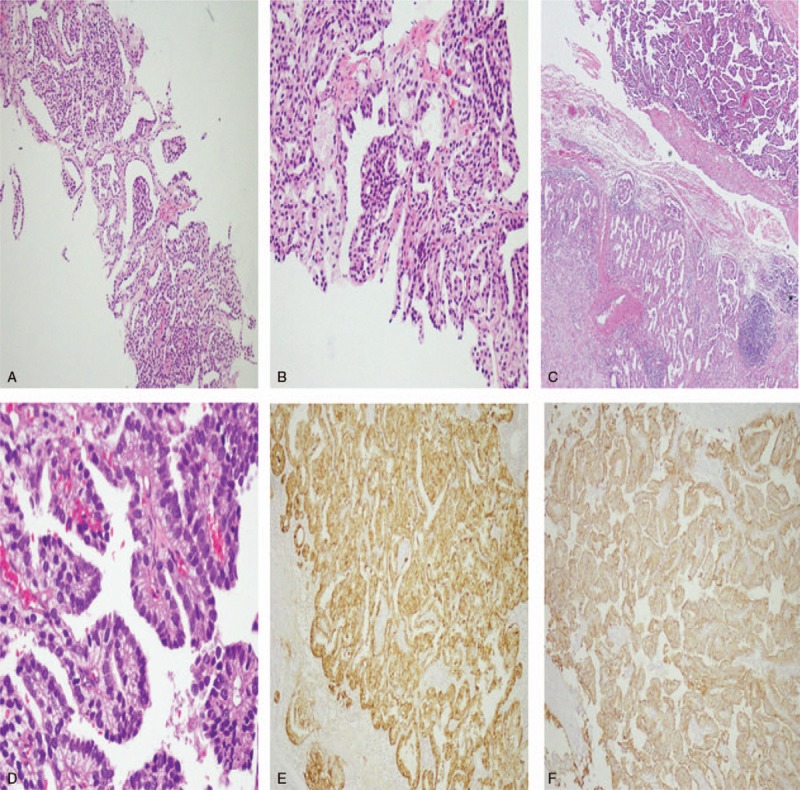
Pathological and immunohistological results. (A) Gleason score was 4 + 3 = 7 (prognostic group III/V), tumor proportion (10/12 mm), and among them, Gleason 4 accounted for about 90% (HE, magnification: 100×). (B) Gleason score was 4 + 3 = 7 (prognostic group III/V), tumor proportion (10/12 mm), and among them, Gleason 4 accounted for about 90% (HE, magnification: 400×). (C) Papillary structure of tumor cells are shown in the upper part of the figure, while glomerular and glandular tubular structures are shown in the lower part (HE, magnification: 400×). (D) Papillary structure of tumor cells (HE, magnification: 400×). (E) Immunohistochemical PSA (+) is shown. (F) Immunohistochemical CK-pan (+) is shown.

The ultrasonographic imaging showed that a hypoechoic mass in the right lower abdomen and the inner echo was uniform, and its size was 7 cm in diameter. The abdominal computed tomography (CT) scan showed a thin-walled cyst located in the lower right area, that was about 7.7 cm in diameter, and CT value was about 16 HU (Fig. 1A). No enhancement was found in the mass on enhanced CT. The mass was adjacent to right psoas major, ureter, and iliac vessels (Fig. 1B and C). Prostate hyperplasia and heterogeneous enhancement of the right portion represented in the CT scanning led to further checks (Fig. 1C). Prostatic central gland and right peripheral zone showed a low intensity on fat-suppressed T2-weighted sequences (Fig. 1D). The capsule of prostate was complete, and extracapsular extension was not found; thus T classification in tumor-node-metastasis staging was known as clinical stage T2c. The intracapsular density and signal was uniform. The fatty space surrounding the lesion was clear. Abdominal CT scanning and magnetic resonance imaging (MRI) of the prostate did not detect other tumors. No infectious findings were found, and there were no infectious findings regarding metastasis on other organs as well. The serum level of prostate specific antigen (PSA) was 131.200 ng/mL. The results of routine urinalysis, hepatitis B, hepatitis C, AIDS virus, syphilis, and coagulation markers were negative. The results of other examinations are presented in Tables 1 and 2. To clarify the nature of the prostatic lesion, transrectal ultrasound guided prostate biopsy was undertaken. The pathological findings of prostate biopsy indicated prostatic adenocarcinoma (Fig. 2A and B). Gleason Score was 4 + 3 = 7. No bone metastases could be found in bone scan with single photon emission computed tomography (SPECT). Combined with the examination results, especially the puncture pathology and PSA results, we can confirm the diagnosis of prostate cancer and exclude other diagnoses as well. The tumor invaded the 2 lobes of the prostate without regional lymph node metastasis and distant metastasis. The preoperative clinical staging of prostate cancer was Stage II (T2cN0M0).

**Table 1:**
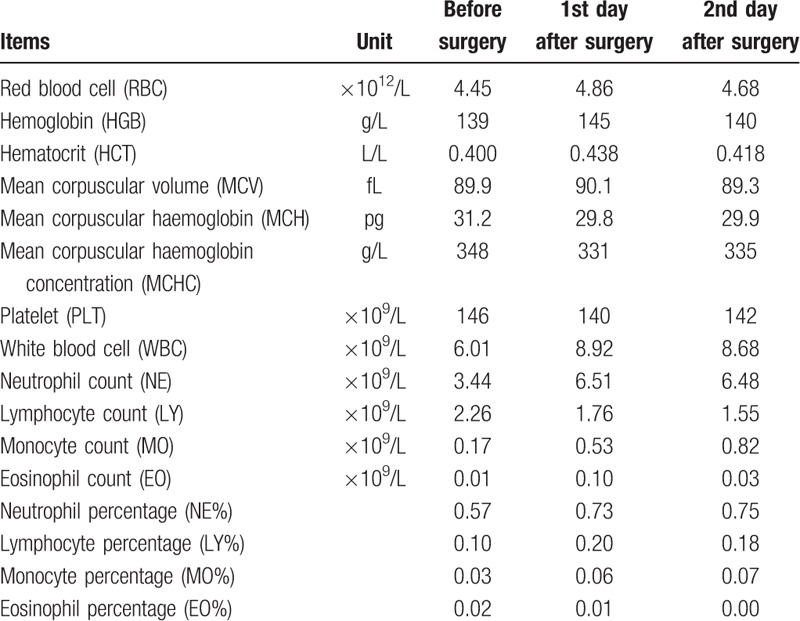
Complete blood count before and after surgery.

**Table 2:**
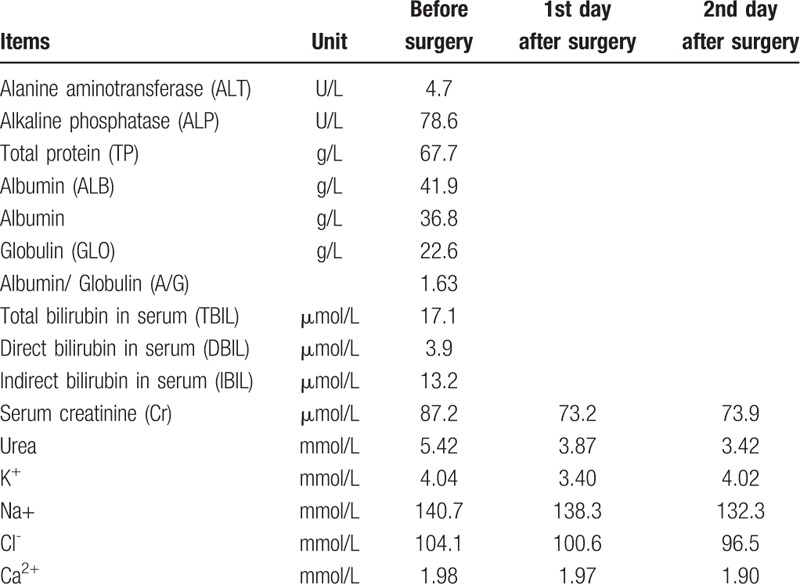
Blood Biochemical Examnation.

It is impossible to determine the benign and malignant nature of cystic lesions, the patient was diagnosed with prostate cancer, and exploration of the lesion was performed after careful consideration. During operation, the cyst, 7.0 cm in size, was located at the bifurcation of right external and internal iliac artery. The cyst appeared complex serious adhesion to surrounding structures. When the cyst stripped during the operation, visible dilute coffee-like-liquid overflowed. This led to an inability to detect the contents of cystic masses. The wall of the cystic lesion was taken as a specimen for pathological examination; the prostate was retained as well.

*Histopathology results*: papillary pattern was visible on the inner wall, and the gland duct and glomerular structure were scattered in the wall (Fig. 2C and D). Immunohistochemistry results demonstrated Calretinin (+), D2-40 (−), Ki-67 (10%+), Vimentin (−), CK-pan (+), CK5/6 (−), WT-1 (−), PSA (+), SALL4 (−), Villin (−), CK20 (−), CK7 (−), PAX-8 (−), and TTF-1 (−). Combined with morphological and immunohistochemical findings (Fig. 2E and F), the result is prone to metastatic prostate adenocarcinoma. In consideration of metastatic lymph nodes and cystic metastasis, the postoperative clinical staging of prostate cancer was stage T2cN1M1c. Radical prostatectomy was no longer appropriate, and androgen deprivation therapy (ADT) was recommended.

According to the results, a 2-year ADT treatment was suggested to the patient after discharge. Subcutaneous injection of goserelin (AstraZeneca UK Limited, London, UK) once a month was combined with 50 mg of bicalutamide (AstraZeneca UK Limited, London, UK) per day. We have given a telephone follow-up of 13 months later. The patient is still taking the above ADT for treatment with no adverse drug reactions. The clinicians believed that the expected effect was achieved. The patient was also satisfied with the effect of the treatment. The patient had satisfactory compliance and tolerance for treatment. In general, every 3 months, the level of PSA was re-examined. The results of local hospital showed that the level of PSA during this period maintained in a proper level (< 4 ng/mL). The latest level of PSA was 0.2 ng/mL recorded on May 5th, 2018. In order to achieve a comprehensive evaluation of the condition, imaging examinations were carried out, including pelvic magnetic resonance, enhanced CT scanning, and bone scanning. No distant metastasis was found as well. The results did not indicate the progression of prostate cancer. Although this was not a long-term imaging evaluation, however, the therapeutic effects could be proved in this period. According to the results of PSA examination, the feasibility of long-term imaging examination was assessed. The restage of the patient was Stage IVB (T2cN0M0). It was revealed that the volume of tumor decreased, no new metastases appeared, and no enlarged lymph nodes were found. The patient achieved a partial response (PR) to the treatment.

## 2 Discussion

In the existing English literature, the solitary cystic metastasis of prostate cancer has never been reported. However, cystic degeneration of prostate cancer is occasionally reported.^[4–7]^ A case of prostate cancer, invading the right seminal vesicle with cystic changes, was the largest cystic variation of the prostate.^[4]^ There have been reports of cystic recurrences following resection of prostate cancer.^[8]^ However, the nature of the cystic lesion is not still clear. Besides, the patient has the diagnosis of prostate cancer. Surgery is also helpful for the differential diagnosis methods of benign or malignant cystic masses.

Recently, the patient had no history of fever and other organ infections. No evidence of infection was found in Tables 1 and 2. In addition, the cystic lesions of the right lower abdomen were not initially considered as infectious diseases.

Necessary vigilance on atypical sites is still required, although the most common metastatic sites of prostate cancer are bone and lymph nodes.^[11]^ Metastasis of prostate cancer has been reported to occur in the ureter, penile, appendix, intracranial, and so on.^[12]^

Around the internal iliac vessels, there is a typical location of pelvic lymph node metastases of prostate cancer. The cystic mass was accompanied with smooth margins, uniformed density, thin wall, and no enhancement. In the CT scanning, no separation and real components solid parts could be found. Solid components and different degrees of enhancement were observed in lymph node metastasis.^[9]^ During histological examination, no residual lymph node tissue was found in the lesion and the wall of the cyst. There was no reliable evidence for lymph node metastasis as well.

Pelvic solitary cystic lesions were associated with cystic lymphangioma. Cystic lymphangioma can occur in any part of the body, however, that frequently occurs in the head and neck, and it may rarely observe in the pelvic.^[10]^ There was no pathological evidence of cystic lymphangioma in the case that we reported. The right part of the pelvic cavity is still an untypical site of cystic lymph node metastasis, cystic lymphangioma, and cystic lymphatic metastasis combined with cystic lymphangioma. According to the location of the lesion, it is also differentiated from the source of the right-side ureter. The lesion was adjacent to the right ureter, while there were no symptoms of ureteral obstruction.

During operation, the fluid content of the ruptured cystic lesion was released. Therefore, we had no way to make cytological and biochemical examination of the liquid content. There are indeed some limitations in evaluating cyst wall. However, the morphological and immunohistochemical findings of the cystic wall can still be identified as metastasis of prostate cancer. Although there are no visible components inside the lesion, it is possible to miss some information. Tracing of lymph tissue or other components could not be fully undertaken, so that there are some deviations in the results.

There has been a study of survival of prostate cancer with different metastasis conditions. The survival time of the patients with different metastases sites ranged from long- to short period, in which the longest and the shortest periods were 3.9 and 2.2 years, respectively, including lymph node, bone, bone with lymph node metastasis, and visceral metastasis.^[13]^ The patient studied in this research achieved no further progress in the disease with regular ADT treatment and close surveillance. Thus, patients with cystic metastasis of prostate cancer may have a satisfactory short-term prognosis. This may be related to the lack of a long-time follow-up. In addition, the absence of metastases from other sites may be another factor. As for prostate cancer, the prognosis of cystic metastasis needs to further follow-up and extension studies.

## 3 Conclusions

When cystic lesions are accompanied with prostate cancer, we should be aware of the possibility of cystic metastasis of prostate cancer. Prostate cancer is a well-known malignancy with protean manifestation. Cystic metastasis of lymph nodes as well as lymphangioma are other conditions which may have a similar picture on scanning as a differential diagnosis. The case herein studied highlighted the diagnostic dilemma, which can arise due to unusual presentation and imaging features of a common malignancy, mimicking some other pathologies.

## Author contributions

**Resources:** Lirong Bi, Zhuo Wang, Yang Zhao, Jiping Wang.

**Writing – original draft:** Bei Zhang.

**Writing – review & editing:** Bingyang Bian, Jiping Wang.
